# Development and Evaluation of Cannabidiol Orodispersible Tablets Using a 2^3^-Factorial Design

**DOI:** 10.3390/pharmaceutics14071467

**Published:** 2022-07-14

**Authors:** Robert-Alexandru Vlad, Paula Antonoaea, Nicoleta Todoran, Emöke-Margit Rédai, Magdalena Bîrsan, Daniela-Lucia Muntean, Silvia Imre, Gabriel Hancu, Lénárd Farczádi, Adriana Ciurba

**Affiliations:** 1 Pharmaceutical Technology and Cosmetology Department, Faculty of Pharmacy, “George Emil Palade” University of Medicine, Pharmacy, Science and Technology of Targu Mures, 540142 Targu Mures, Romania; robert.vlad@umfst.ro (R.-A.V.); nicoleta.todoran@umfst.ro (N.T.); emoke.redai@umfst.ro (E.-M.R.); magdalena.birsan@umfiasi.ro (M.B.); adriana.ciurba@umfst.ro (A.C.); 2 Drug Industry and Pharmaceutical Biotechnology Department, Faculty of Pharmacy, “Grigore T. Popa” University of Medicine and Pharmacy from Iasi, 700115 Iasi, Romania; 3 Analytical Chemistry and Drug Analysis Department, Faculty of Pharmacy, “George Emil Palade” University of Medicine, Pharmacy, Science and Technology of Targu Mures, 540142 Targu Mures, Romania; daniela.muntean@umfst.ro (D.-L.M.); silvia.imre@umfst.ro (S.I.); 4 Chromatography and Mass Spectrometry Laboratory, Centre for Advanced Medical and Pharmaceutical Research, “George Emil Palade” University of Medicine, Pharmacy, Sciences and Technology of Targu Mures, 38 Gheorghe Marinescu Street, 540142 Targu Mures, Romania; lenard.farczadi@umfst.ro; 5 Pharmaceutical and Therapeutic Chemistry Department, Faculty of Pharmacy, “George Emil Palade” University of Medicine, Pharmacy, Science and Technology of Targu Mures, 540142 Targu Mures, Romania; gabriel.hancu@umfst.ro

**Keywords:** orodispersible tablets, cannabidiol, pharmacotechnical evaluation, full factorial design, co-processed excipients

## Abstract

Orodispersible tablets (ODTs) are pharmaceutical formulations used to obtain fast therapeutic effects, usually recommended for geriatric and pediatric patients due to their improved compliance, bioavailability, ease of administration, and good palatability. This study aimed to develop ODTs with cannabidiol (CBD) phytocannabinoid extracted from *Cannabis sativa* used in the treatment of Lennox–Gastaut and Dravet syndromes. The tablets were obtained using an eccentric tableting machine and 9 mm punches. To develop CBD ODTs, the following parameters were varied: the Poloxamer 407 concentration (0 and 10%), the type of co-processed excipient (Prosolv^®^ ODT G2—PODTG2 and Prosolv^®^ EasyTab sp—PETsp), and the type of superdisintegrant (Croscarmellose—CCS, and Soy Polysaccharides—Emcosoy^®^—EMCS), resulting in eleven formulations (O1–O11). The following dependent parameters were evaluated: friability, disintegration time, crushing strength, and the CBD dissolution at 1, 3, 5, 10, 15, and 30 min. The dependent parameters were verified according to European Pharmacopoeia (Ph. Eur.) requirements. All the tablets obtained were in accordance with quality requirements in terms of friability (less than 1%), and disintegration time (less than 180 s). The crushing strength was between 19 N and 80 N. Regarding the dissolution test, only four formulations exhibited an amount of CBD released higher than 80% at 30 min. Taking into consideration the results obtained and using the *Modde 13.1* software, an optimal formulation was developed (O12), which respected the quality criteria chosen (friability 0.23%, crushing strength of 37 N, a disintegration time of 27 s, and the target amount of CBD released in 30 min of 99.3 ± 6%).

## 1. Introduction

Orodispersible tablets (ODTs) are modern pharmaceutical formulations easily accepted by patients with a maximum disintegration time according to European Pharmacopoeia 10th edition (Ph. Eur. 10) of 3 min [1,2,3]. An advantage of this pharmaceutical formulation is related to the fact that no water intake is needed during administration [4,5,6]. In the past, the targeted populations for the OD formulations were pediatric and geriatric patients with Parkinson’s disease, schizophrenia, or gastroesophageal reflux, but recently, ODTs are used for a large category of patients due to their numerous advantages:

- do not necessitate chewing;
- disintegration takes place directly in the mouth;
- pleasant taste;
- increased stability compared to solutions, emulsions, and suspensions;
- can be manufactured as controlled released pharmaceutical formulations;
- high amounts of Active Pharmaceutical Ingredient (API) can be used;
- ability to confer the advantages of a liquid formulation in a solid form;
- the therapeutic effect occurs fast [7,8,9,10,11].

Several technologies for developing ODTs have been patented, such as Orasolv^®^, Durasolv^®^, WOWTAB^®^, Flashtab^®^, Zydis^®^, Quicksolv^®^, and Lyoc^®^ [12,13,14,15]. The manufacturing methods are complex, needing multiple steps that are easily prevailed in the pharmaceutical industry. One of the methods often used is the direct compression method. This method is the most convenient due to the low costs and the efficacy in developing the final product [14].

Cannabidiol (CBD) represents one of the major components alongside tetrahydrocannabinol (THC) that can be found in *Cannabis* species [16,17]. As a result of its complex mechanism of action, it has the potential to be used in different pathologies; CBD has a low affinity for the CB_1_ and CB_2_ receptors acting as an antagonist on GPR_55_ receptors, presents a reversed agonist effect on GPR_3_, GPR_6_, and GPR_12_ receptors, and partially agonist on the 5HT_1-A_, which together might explain CBD’s potential antidepressant, antianxiety, and neuroprotective effects [18,19,20,21,22]. Currently, two products containing CBD have been approved by Food and Drug Administration (FDA) and European Medical Agency (EMA): Epidiolex^®^ and Sativex^®^ [23,24,25]. Epidiolex^®^ is used for patients with Lennox–Gastaut or Dravet syndromes as second-line antiseizure medication (ASM) [26,27,28,29]. Sativex^®^ contains both CBD and THC, a fact that makes it unavailable in many European states, due to the strict laws regarding THC use [25]. However, there are a large number of products containing CBD marketed as dietary supplements, as the tendency of using this type of product is increasing due to the changes in the regulatory environment and perceptions of their health benefits.

Because of the small number of approved and available pharmaceutical formulations with CBD, this study aimed to develop CBD ODTs that can be used for pediatric patients to treat drug-resistant epilepsy.

For developing CBD ODTs the following criteria must be considered [3,30]:

- disintegration in the mouth has to occur faster than 3 min, taking into consideration the stipulation of Ph. Eur. 10, while the United States Pharmacopoeia 44 (USP 44) presents an even shorter disintegration time (<30 s) [3,31];
- the API has to be compatible with the used excipients;
- the ODT has to be easy to handle;
- the palatability properties have to be taken into consideration;
- after oral administration no waste or small waste should be found in the oral cavity;
- the API should not be influenced by the temperature and humidity conditions and their variation [30];
- the ability to trespass the buccal mucosa;
- partly unionized at the buccal cavity pH;
- the capacity of passing through the gastrointestinal epithelium;
- a long half-life—in this case, CBD has a half-life of 24 h;
- increased stability in water and saliva [31,32,33].

It has been widely acknowledged since the introduction of Quality-by-Design (QbD) concepts that pharmaceutical product quality should be developed and created according to an experimental plan during the manufacturing process. Traditionally, pharmaceutical product development and optimization have been done by applying a “one factor at time” (OFAT) approach, in which one of the variables is modified within a reasonable range, while the rest remain unchanged. OFAT approach requires a large number of experiments and does not allow evaluation of the interaction that exists between the factors, which may lead to inadequate conduction of the development and optimization. To overcome these problems, design of experiment (DoE) strategies are recommended to obtain better results with few numbers of experiments [34,35,36,37,38]. For developing CBD ODTs, a two-level full factorial design with three variables was used. Using *Modde 13.1.* software, a final optimized formulation was obtained that considers the independent factors, while also taking into consideration targeted answers from the dependent factors.

## 2. Materials and Methods

### 2.1. Powder Characterization

The bulk and tapped densities (D_a_, D_t_) were calculated in accordance with chapter 2.9.34 from the Ph. Eur. 10. The powders belonging to the 11 formulations proposed were characterized in terms of Carr Index (CI) [31], Hausner Ratio (HR) (2.9.36, Ph. Eur. 10), and porosity (ε) (2.9.32, Ph. Eur. 10) [3]. The tapped density was determined using an electronic densimeter (MZ-P3000 electronic densimeter, China).

### 2.2. Experimental Design Optimization

The CBD ODT (O1-O11) development utilized a 2^3^ full factorial design with *Modde 13.1* Software (Umetrics, Sweden). The composition of the eleven formulations (eight plus three central points) can be found in Table 1, while the independent variables can be found in Table 2. The answers (dependent variables) are presented in Table 3.

### 2.3. CBD ODT Manufacturing Steps

The CBD ODTs were prepared according to the matrix of the DoE (Table 1). The average weight of a tablet was set to 200 mg containing 10 mg of CBD. The independent factors (Table 2) varied as follows: co-processed excipient (PETsp and PODTG2), superdisintegrant type (CCS and EMCS), and PLX407 amount (0 and 10%), resulting in eight formulations with three central points that were developed taking into consideration the matrix generated by *Modde 13.1* software. The tablets were obtained using an eccentric tableting machine, Korsch 0 (Berlin, Germany), with punches of 9 mm. For each formulation, the inferior position of the lower punch was adjusted because of the different densities and porosities of the obtained mixtures. The powders were mixed with respect to the rule of blending powders (increasing quantities, descending densities), with the flavor (banana flavor) being added in the end. Then, besides the independent variables presented in Table 3, other parameters were verified: average weight, ODT diameter, ODT radius, and ODT thickness.

### 2.4. Evaluation of the Dimensional Parameters Belonging to the CBD ODTs Developed

Average weight was determined using an analytical balance (Kern, Berlin, Germany). The ODT diameter, radius, and thickness were determined using an electronic micrometer (Yuzuki, New Delhi, India).

### 2.5. Evaluation of the Dependent Parameter

Four dependent parameters were evaluated: friability, crushing strength, disintegration time, and the amount of CBD released during the 30 min dissolution test.

#### 2.5.1. Friability

This parameter was evaluated according to requirements stipulated by Ph. Eur. 10, using 20 tablets for each formulation [3]. To establish this parameter, the TFUT3 Tablet Four Usage Tester Model (Biobase, Jinan, China) was used. The apparatus drums rotated 100 times for four minutes (25 rot/min). In the end, the loss mass was expressed in percentages, with the maximum limit admitted being 1% [3].

#### 2.5.2. Crushing Strength

The crushing strength was obtained using the same four functions apparatus used to verify friability—TFUT3 Tablet Four usage TesterModel—(Biobase, China) [3,33]. For this, ten CBD ODTs from each formulation were tested and their crushing strength was expressed in N.

#### 2.5.3. Disintegration Time

The disintegration ability was determined using the TFUT3 Tablet Four Usage TesterModel—(Biobase, China) at a temperature of 37 ± 1 °C [3,31,33]. The disintegration time was impartially evaluated using the disintegration method with disks on six tablets from each proposed formulation.

#### 2.5.4. In Vitro Dissolution Test

The amount of CBD released from the ODTs was evaluated using a UHPLC method previously validated according to ICH guidelines [39]. The apparatus used was an Erweka Two with paddles (Erweka, Langen, Germany), using a rotation speed of 75 rpm. The dissolution media consisted of 1000 mL of phosphate buffer and 0.052 M sodium lauryl sulphate with a pH of 6.8. Then, 2 mL aliquots were taken at 1, 3, 5, 10, 15, and 30 min and were replaced with 2 mL of fresh dissolution media.

### 2.6. Development and Validation of a UHPLC Method for the Quantification of the Released CBD

#### 2.6.1. Reagents

In the UHPLC procedure, the following reagents and reference substances were used: CBD (99.5%, Trigal Pharma, Vienna, Austria), acetonitrile (ACN) (SLW Chemicals, Muskegon, MI, USA), and purified water obtained using a Direct Q3 System.

#### 2.6.2. Instruments

To evaluate the amount of CBD released a Shimadzu UHPLC Nexera Series was used coupled with an UV-VIS detector (photodiode array (PDA) type) (Shimadzu, Kyoto, Japan). An InfinityLab Poroshell 12 EC-C18 column (3 × 100 mm, particle size of 2.7 µm) (Agilent, Santa Clara, CA, USA), was utilized. The injection volume was set to 10 µL while the flow rate was 1 mL/min.

#### 2.6.3. Stock Solution and Quality Control Samples

The stock solution concentration was selected to be 1 mg/mL and was obtained by dissolving 10 mg of CBD in 10 mL of ACN. Five concentrations were selected for the calibration curve (0.5 µg/mL, 1 µg/mL, 2.5 µg/mL, 5 µg/mL, and 10 µg/mL), adapting the calibration range to the concentration domain that might be released in the total volume of the dissolution media of 1000 mL (0–10 µg/mL). The preparation of the concentrations used in calibration was made by diluting the stock solution with the dissolution media.

#### 2.6.4. Validation Criteria

To verify the analytical performance of the method, the following parameters were evaluated: linearity, carry-over, selectivity, accuracy, and precision.

## 3. Results and Discussion

### 3.1. Powder Evaluation

The porosity (Table 4) ranged from 0.18 (O1) to 0.37 (O4). Usually, a low porosity results in better compressibility or an improvement in this parameter; the same being applied in the case of CI. Further, with the help of HR, the flow character can be determined. In this manner, it was observed that O1 presented good flowability (value < 1.25), while the majority of powders corresponding to the formulation proposed presented values between 1.25–1.5 (O2, O3 O5–O11). The following mixtures are considered passable according to Ph. Eur. 10: O2, O3, O5, O7, O9, O11; while O1 has fair flow properties. The other formulations (O6, O8, O4) have poorer flow characteristics according to the scale of flowability from the Ph. Eur. 10. To improve the flowability, a lubricant might be added or the amount of lubricant can be increased, but this modification must be done considering the compressibility, which might decrease through this modification.

### 3.2. Evaluation of the Qualitative and Quantitative Dimensional Parameters Belonging to the CBD ODTs

To establish the dimensional parameters of the CBD ODTs, the following parameters were determined: average weight, diameter, average radius, and thickness; the results of the previously mentioned dimensional parameters are found in Table 5.

#### 3.2.1. Uniformity of Mass

The average weight fitted the admitted limits regarding uniformity, and all the tablets presented a standard deviation of less than 7.5 % in comparison with the average mass of each formulation (Table 5). The maximum deviations were (−6.3533%—O3) and (6.5121%—O5). Taking into consideration the fact that the maximum limit is ±7.5 %, we concluded that all the developed tablets correspond to the stipulations of the average mass uniformity from Ph. Eur. 10 [3].

#### 3.2.2. CBD-ODTs Average Radius, Diameter, and Thickness

In the case of the developed CBD-ODTs, the diameter of the tablets was between 9.032 mm for O10 and 9.122 mm for O5 formulation (Table 5). Note that the values obtained are slightly larger than the punch diameter. In terms of percentage, the punch diameter was exceeded, with 1.35% for O5 and 0.35% for O3. The CBD-ODTs radius was calculated using Equation (1).

r = D/2(1)

where,

r = tablets radius in mm,

D = tablet diameter in mm.

The values obtained for the radius were between 4.521 mm (for O3) and 4.561 mm (for O5), and the deviations had values between 0.002 and 0.012 mm.

### 3.3. Developing CBD ODTs—2^3^ Full Factorial Design

The aim of this study was the development, evaluation, and optimization of a CBD-ODT formulation using a two-level full factorial design. A full factorial DoE was selected because this type of design is the most powerful screening design, allowing the estimation of the main effects of input factors and their interactions on output responses. For two-level full factorial designs, the number of experiments required is 2^k^, where k is the number of input factors to be explored (in our case k = 3) [40]. The results for the selected dependent variables can be found in Table 6.

All the formulations passed the friability test (Y1) (Table 6), with all evaluated formulations presenting values smaller than 1%. The lowest value was registered in the case of O5 formulation (0.24%).

Regarding the crushing strength—Y2 (Table 6), it can be noticed that the tablets with PETsp tend to be more resistant. For O4, a crushing strength of over 80 N was registered, while the ODTs where PODTG2 was used (O11) presented a smaller crushing strength of 53.13 N.

The values usually accepted for the crushing strength are between 35–75 N, but if an ODT with a higher crushing strength and a good disintegration time is developed, higher values for this mechanical property are accepted. Also, an increased value of the crushing strength does not imply special storage conditions, a fact that can be considered an advantage.

For the tablets with a smaller crushing strength, the use of special blisters that prevent future mechanical shocks which might conduct to breaking/crushing/fractionation of the tablet is recommended. The ODTs with PETsp and without PLX407 presented the highest values regarding the crushing strength.

Four formulations can be included in the proposed interval of 35–75 N (O1, O2, O10, O11), six of them exhibited values lower than 35 N (O3, O5, O6, O7, O8, O9), while one formulation presented a crushing strength higher than the maximum proposed value of 75 N (O4).

Other studies that included the crushing strength determination of different types of ODTs with various APIs presented the following results: for the ODTs with prednisolone, an average value in a proposed interval (35–75 N) of 62.6 N was registered, while in the case of paracetamol, ODT crushing strengths between 63–73 N were recorded for all 36 formulations evaluated [41,42].

From the disintegration time (Y3) point of view (Table 6), all the formulations are according to the Ph. Eur. 10 stipulations with a disintegration time smaller than 180 s [3]. The smallest disintegration time was obtained in the case of O2 and O4 formulations; eight formulations (O1, O2, O3, O4, O6, O9, O10, and O11) presented a disintegration time of less than 30 s, which is in accordance with the USP 44 stipulations (disintegration time smaller than 30 s) [31]. The formulation with the highest disintegration time contained PODTG2 and 10% PLX407, indicating a possible decrease in the disintegration time. As in the case of O8, a concentration of 10% PLX407 led to a higher disintegration time; however, in this case, another co-processed excipient was also used (PETsp).

Taking into consideration the results published in the literature, the disintegration time is often influenced by the compression pressure [43,44,45], disintegrant type [46,47,48], and the amount of disintegrant [49,50], these being the main factors responsible for the disintegration time variation. The disintegration time represents a previous step of the dissolution test, with an increased value of the disintegration time implying a slower release of the API, while a fast disintegration time often suggests a faster therapeutic effect.

Evaluation of the CBD quantity released in 30 min showed that four formulations released a concentration of CBD higher than 80%. The formulations that released amounts of API smaller than 80% were O1–O7. The fastest CBD release was recorded in the case of O10: in 1 min, 67.77% of CBD was released. A slower release was registered in the case of O1 (48.46%), O2 (30.16%), O3 (21.45%), O4 (29.55%), O5 (53.05%), O6 (76.33%), O7 (63.87%), formulations that do not fulfill USP requirements of 80% API released at 30 min.

If the critical threshold is lowered to 5 min, it can be observed that in the case of O11, over 80% of API was released.

**Figure 1:**
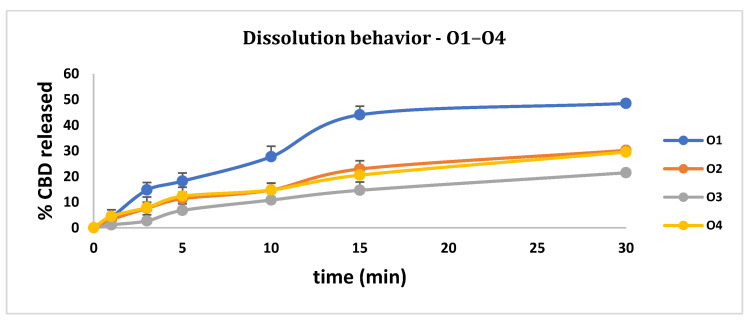

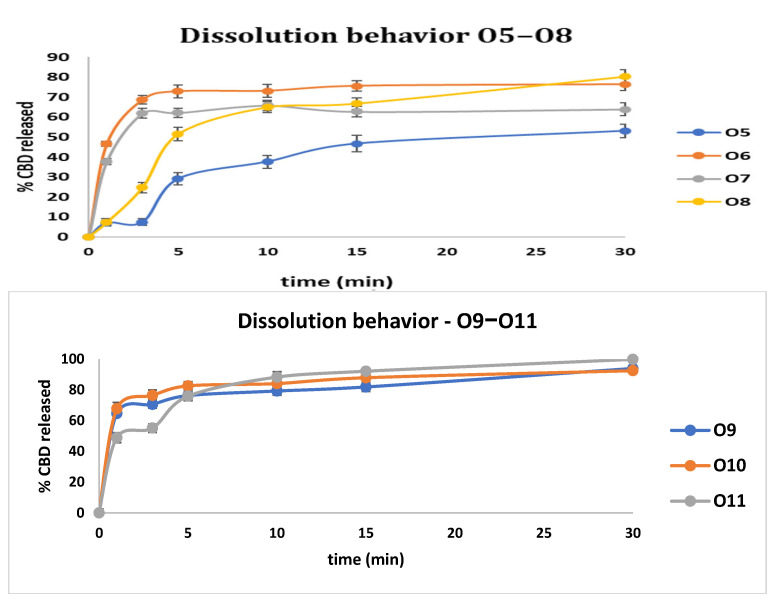
Dissolution profiles of the CBD ODT formulations.

A factor that might cause a slower release of the API is the amount of PLX407, which might be responsible for the slower release of the API in the formulations O5, O6, and O7. The results regarding the dissolution behavior are presented in Figure 1.

All eight formulations plus three central points were evaluated using the *Modde 13.1* software during the statistical evaluation of the results. Data processing was accomplished using the partial least squares method, analyzing R^2^ and Q^2^ using Analysis of Variance (ANOVA) analysis. R^2^ indicates the variation of the response as explained by the model, while Q^2^ indicates the variation of the response that can be predicted by the proposed model. In both cases, the values were in general higher than 0.5, with proximity to 1 indicating a good model with increased prediction [51,52]

**Figure 2:**
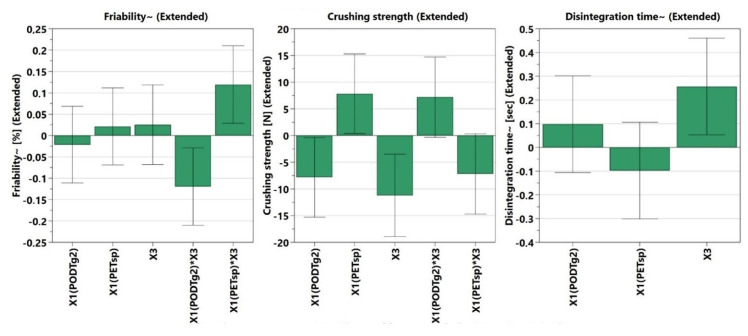

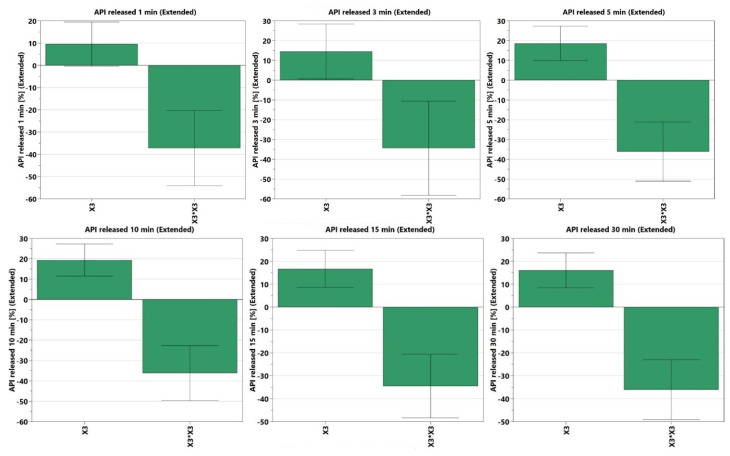
The influence of the independent variables on the dependent factors (Y1–Y9).

Figure 2 and Table S2 (Supplementary Materials) present the formulation factors and the way these influence the dependent factors considered in this study.

Friability (Y1) was influenced by the type of co-processed excipient used (Figure 2). This parameter was negatively influenced when PLX407 and PETsp were used. When PODTG2 and PLX407 were used, a positive influence can be observed. The interaction of PODTG2*X3 was conducted to a lowered friability compared to PETsp*X3, where increased values of the friability were obtained. In this case, the value must be as low as possible. The disintegration time (Y3) was influenced as follows: disintegration time increased based on the amount of PLX407 or PODTG2 that was used, while disintegration time decreased when the co-processed excipient (PETsp) was used (Figure 2). In the case of the co-processed excipient, the disintegration behavior can be explained through the composition. The main filler in PODTG2 is MNT, an excipient that can increase the disintegration time, while in the case of PETsp, the filler used is CelMC, an excipient known for its good disintegration properties (Figure 2).

Crushing strength (Y2) was influenced negatively (decreased) by the use of PLX407. Also, the use of PODTG2 and the interaction of X1(PETsp)*X3 resulted in a decreased crushing strength, while PETsp and the interaction between X1(PODTG2) * X3 influenced the crushing strength positively, by increasing it (Figure 2).

The dissolution profiles are presented in Figure 1 for O1–O11. The API released was investigated at 1 (Y4), 3 (Y5), 5 (Y6), 10 (Y7), 15 (Y8), and 30 min (Y9). In all six evaluations, the only statistically significant factors were X3 and X3*X3. X3*X3 could be evaluated because of the presence of the central points where the concentration of PLX407 was 5%; the concentrations of PLX407 considered in this experiment were 0 and 10%. The in vitro dissolution profile is influenced positively by the X3 amount, but the interaction (X3*X3) might result in an extended release (Figure 2).

**Figure 3:**
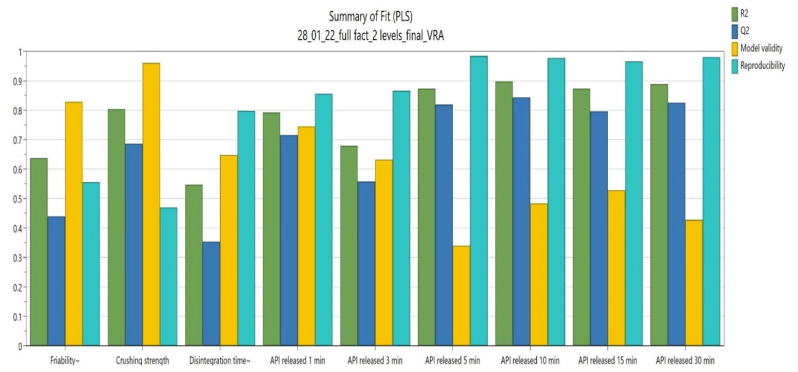
Summary of fit.

The results concerning the CBD ODT development using a 2^3^ full factorial design are presented in Figure 3 and Table 7, including the following parameters: R^2^, R^2^ adj (adjusted R^2^), Q^2^, SDY (standard deviation of the response), validity, RSD (residual standard deviation), N (number of experiments), and reproducibility.

Via the ANOVA test, we can conclude that the results are due to the formulation factor or present a natural variation. For eight out of nine responses, the *p*-value had a value lower than 0.05, with only Y1 presenting a slightly increased value of 0.057.

R^2^ (Figure 3) presented values higher than 0.8 for five of the evaluated answers, and only Y3 presented a smaller value (0.55). Q^2^ (Figure 3) exhibited values between 0.35 and 0.84, with the smaller value again being attributed to the Y3 answer. Validity showed values higher than 0.25 for all answers (values smaller than 0.25 usually indicating a statistically significant model problem, an incorrect model, or a transformation problem), while the reproducibility presented values higher than 0.5 for eight answers and seven out and nine exhibiting values higher than 0.8 (Figure 3). Considering the obtained data, we can conclude that the chosen model fits the proposed answers.

In a study conducted by Abed et al., in which diazepam ODTs were developed (using three types of disintegrants: SSG, Ac-Di-Sol, and crospovidone in a concentration of 10%), the results of the friability were also according to Ph. Eur. stipulations, with one exception: for the composition that used crospovidone and camphor, the excipients caused an increased friability. In the same study, the dissolution behavior was studied in comparison with an already available conventional tablet, as in our study. The diazepam ODTs presented API between 90–100% at 10 min for the three selected formulations: F4—crospovidone and ammonium bicarbonate, both 10%, F6—crospovidone 10%, ammonium bicarbonate 15%, and F7—crospovidone 10% and ammonium bicarbonate 20%. Camphor and ammonium bicarbonate served as subliming agents [53]. In another study with the same objective of developing ODTs but with fluoxetine as the API, conducted by Marzouk et al., four superdisintegrants were used: crospovidone, CCS, SSG, and indion, all of them varied on three levels (2, 3, and 4%). In the case of the fluoxetine ODT, the friability had values very close to 0.5% for all formulations and the tablet hardness varied between 31 N to 45 N. The largest crushing strength was seen in the formulation that used SSG in a higher amount (4%), while the lowest was observed in the case of the formulation where indion was used (3%). Fast disintegration times of lower than 12 s were obtained in all the cases, a fact that can be explained by the use of Avicel^®^, an excipient that contains CelMC [54]. In a study that aimed to develop glibenclamide and metformin ODTs where a full factorial design 3^2^ was used, the amount of water and pregelatinized starch were considered the independent variables. Friability lower than 1% was recorded, with disintegration times between 33–91 s. The amount of glibenclamide released at 30 min was between 84–91%, while in the case of metformin, 89–95% of API was released. Both independent variables had a significant impact on tablets’ properties, with the pregelatinized starch exhibiting a pronounced effect on the disintegration behavior [55]. The results obtained in our study are comparable with the results available in the literature in terms of friability, disintegration time, and dissolution behavior. The crushing strength of the tablets developed in the previously mentioned articles had the following maximum values: for the metformin and glibenclamid ODTs, the result (99.53 N) was close to that of the O4 formulation from this study; for the diazepam ODTs, the maximum crushing strength recorded was 36.28 N; for the fluoxetine ODTs, the maximum crushing strength recorded was 46 N [53,54,55].

### 3.4. Development and Validation of a UHPLC Method for the Quantification of the Released CBD

The separation of the analytes was conducted at a temperature of 23 ± 2 °C. Several mobile phases were evaluated, and a good retention time was obtained while using 30% water and 70% ACN (2.8 min); this mobile phase was considered suitable for the evaluation of CBD released. The optimal wavelength was 225 nm, the flow rate was set at 1 mL/min, and the injection volume was 10 µL. The retention time in optimal conditions was 2.8 min, which can be considered fast. While using a blank sample (dissolution media) or the ODT without CBD (composition in Table S1), no interferences were observed and no carry-over was noticed after three solutions of 0.5, 2.5, and 10 µg/mL were injected in between a blank sample. The accuracy and precision were fulfilled by the developed method. The details regarding the UHPLC method developed can be retrieved in the Supplementary Materials Section (selectivity—Figure S1, carry-over—Figure S2, accuracy, and precision—Figure S3).

We compared our results with other studies using chromatographic methods to evaluate the amount of CBD in different matrixes. Ravula et al., proposed a similar method in terms of efficiency and analytical performance [56], while Zgair et al., published a method with a higher retention time of 8.3 min [57]. Another study conducted by Mandrioli et al., reports a retention time of 4.05 min, higher than the retention time of our method [58]. In a study conducted by Grafinger et al., the CBD and THC amount in oils from Switzerland have been evaluated; a retention time higher than six minutes was obtained [59].

### 3.5. CBD ODT Optimization

According to the ICH Q8 Guidelines, in the case of ODTs, some target profile parameters influence the effectiveness of the proposed pharmaceutical product [39].

In the case of CBD-ODTs, it is necessary to take into consideration the following criteria:

- Pharmaceutical formulation: orodispersible tablets;
- Administration route: oral;
- API amount: 10 mg CBD;
- Therapeutic use: Lennox–Gastaut and Dravet Syndromes [28,60];
- Packaging: PVC blister;
- Presentation: white tablet, with intact, fine margins with a diameter of 9 mm;
- API identification: CBD;
- Uniformity of content: 10 mg ± 15% [3,31];
- Friability < 1% [3,31,41,61];
- Dissolution: more than 80% at 30 min [31].

The analysis resulted in the following optimal formulation, taking into consideration the following restraints:

- Friability < 1%;
- Good crushing strength;
- Minimum disintegration time;
- Target amount of CBD is 100% released by 30 min.

The composition of the optimal formulation is presented in Table 8.

**Figure 4:**
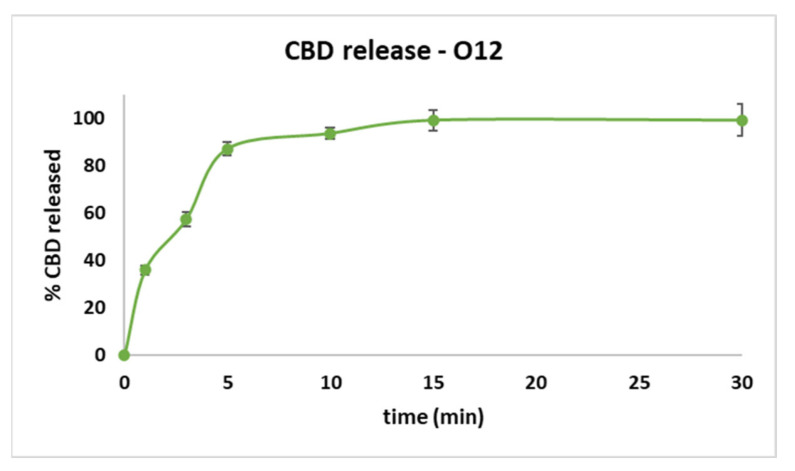
The amount of API released for O12.

The results of the optimal formulation considering the dependent parameters and the estimated values can be found in Table 8 and Figure 4. The dimensional parameters (average weight 0.1979 ± 0.0032 g), ODT diameter (9.002 ± 0.007 mm) and radius (4.501 mm), and tablet thickness (2.694 ± 0.077 mm) were determined for O12. The radius, diameter, and thickness of the CBD ODT presented values are close to those determined theoretically.

The optimized formulation respects the experimental plan regarding friability, crushing strength, and disintegration behavior. As it can be noticed in Table 9, the crushing strength is better than that predicted by the software; also, the disintegration time has a decreased value in comparison with what was predicted. Friability is less than 1%, as in the other CBD ODTs developed, respecting the stipulations of Ph. Eur. and USP. In the case of crushing strength, an advantage is the fact that the CBD ODTs do not need special storage conditions.

The releasing profile of the O12 (Figure 4) showed smaller values regarding the amount of CBD released in the first three minutes, but between 5- and 30-min higher values than the ones predicted were recorded. The selected formula respects the stipulations of USP, with over 80% of API being released at 5 min and almost 100% at 30 min. The results obtained are close to the one predicted by *Modde 13.1* software, so it can be concluded that the results are fitting the model chosen.

## 4. Conclusions

The O1-O11 powders were characterized in terms of porosity, CI, and HR, being included in a preformulation study through which the influence of the porosity on the dependent parameters was observed.

All the proposed formulations meet the friability criteria of < 1%. Crushing strength values higher than 75 N were recorded but the dissolution behavior was not as expected, even though the disintegration time was low. The crushing strength behavior of the formulations presents values between 19–80 N, which indicates influence from both quantitative and qualitative factors that were taken into consideration. The tablets that contained PODTG2 presented a lower crushing strength, which is explained by the presence of MNT, an excipient responsible for the crushing strength decrease.

The critical parameters represented by the disintegration time are fulfilled by all the proposed formulations according to the Ph. Eur. 10 stipulations (<180 s), while eight of the formulations satisfy the more rigorous stipulations of the USP (<30 s).

Evaluation of CBD released from the orodispersible formulations showed that four out of eleven formulations presented an amount of API released higher than 80% at 30 min; also, the optimal formulation presented a value of almost 100% CBD released at 30 min. Increased concentrations of PLX407 might result in slower release of the API. The optimal formulation exhibited over 80% of CBD released after five minutes.

It has been noticed that an increased value of PLX407 might result in a lower crushing strength and negatively influence CBD release.

The developed UHPLC method used to evaluate the amount of CBD released uses a simple mobile phase and is fast (retention time of 2.8 min) and efficient, presenting both accuracy and precision. Also, the method developed respects the current ICH guidelines regarding the development of analytical methods; the analytical method performances were verified in terms of linearity, carry-over, and selectivity.

A new formulation for pediatric use was developed that could treat Lennox–Gastaut or Dravet Syndromes, where other ASMs did not provide the expected results.

## Figures and Tables

**Table 1:** The CBD ODTs proposed and their composition.

Components	Formulation Code										
	O1	O2	O3	O4	O5	O6	O7	O8	O9	O10	O11
CBD ^1^	10	10	10	10	10	10	10	10	10	10	10
PETsp ^2^	-	168.44	-	168.44		148.44	-	148.44	-	-	
PODTG2 ^3^	168.44	-	168.44	-	148.44	-	148.44	-	158.44	158.44	158.44
CCS ^4^	-	-	7.5	7.5	-	-	7.5	7.5	-	-	-
EMCS ^5^	7.5	7.5	-	-	7.5	7.5	-	-	7.5	7.5	7.5
SRB ^6^	6.56	6.56	6.56	6.56	6.56	6.56	6.56	6.56	6.56	6.56	6.56
MNT ^7^	5	5	5	5	5	5	5	5	5	5	5
PLX407 ^8^	-	-	-	-	20	20	20	20	10	10	10
BFL ^9^	2.5	2.5	2.5	2.5	2.5	2.5	2.5	2.5	2.5	2.5	2.5
Final mass (mg)	200	200	200	200	200	200	200	200	200	200	200

^1.^ CBD—cannabidiol (Trigal Pharma, Vienna, Austria, powder—99.5% purity) ^2.^ Prosolv^®^ EasyTab sp—PETsp (JRS PHARMA, Germany), ^3.^ Prosolv^®^ ODTG2—PODTG2 (JRS PHARMA, Rosenberg, Germany), ^4.^ Vivasol^®^ Sodium Croscarmellose—CCS (JRS PHARMA, Rosenberg, Germany), ^5.^ Emcosoy^®^ STS IP—EMCS (JRS PHARMA, Rosenberg, Germany), ^6.^ Sorbitol—SRB (Roth, Karlsruhe, Germany), ^7.^ Mannitol—MNT (VWR Pharmaceuticals, Rosny-sous-Bois, France), ^8.^ Poloxamer 407—PLX407 (Sigma Aldrich, St. Louis, MO, USA) ^9.^ Banana flavor—BFL (Gartenfeld, Mainz, Germany).

**Table 2:** Independent parameters included in the study.

Independent Factors		Level		
Factor 1 Co-processed Excipient Type	X1	−1 PODTG2	0 PODTG2 ^a^	+1 PETsp
Factor 2 Superdisintegrant type	X2	−1 EMCS	0 EMCS ^a^	+1 CCS
Factor 3 PLX407 concentration	X3	−1 0%	0 5%	+1 10%

^a^ The PODTG2 and EMCS were chosen by the software to be the qualitative central points parameters.

**Table 3:** The evaluated answers (dependent parameters).

Responses	Name	Measuring Unit	Admitted/Targeted Value
Y1	Friability	%	<1%
Y2	Crushing strength	N	35–75 N
Y3	Disintegration time	s	<180 s
Y4	CBD released—1 min	%	Maximized
Y5	CBD released—3 min	%	Maximized
Y6	CBD released—5 min	%	Maximized
Y7	CBD released—10 min	%	Maximized
Y8	CBD released—15 min	%	Maximized
Y9	CBD released—30 min	%	100%

**Table 4:** The powder evaluation for O1–O11 CBD ODTs.

Evaluated Parameter	Formulation Code										
	O1	O2	O3	O4	O5	O6	O7	O8	O9	O10	O11
Code	Average Value ± SD										
D_a_	0.60 ± 0.016	0.40 ± 0.009	0.63 ± 0.006	0.41 ± 0.002	0.48 ± 0.009	0.36 ± 0.005	0.5 ± 0.002	0.38 ± 0.012	0.51 ± 0.01	0.5 ± 0.005	0.51 ± 0.021
D_t_	0.74 ± 0.022	0.52 ± 0.011	0.80 ± 0.013	0.65 ± 0.034	0.65 ± 0.0017	0.51 ± 0.010	0.67 ± 0.018	0.53 ± 0.011	0.69 ± 0.019	0.69 ± 0.019	0.69 ± 0.019
ε	0.18 ± 0.041	0.24 ± 0.002	0.22 ± 0.004	0.37 ± 0.029	0.24 ± 0.034	0.29 ± 0.004	0.25 ± 0.051	0.27 ± 0.007	0.26 ± 0.005	0.27 ± 0.013	0.26 ± 0.01
CI	18.18 ± 4.14	24 ± 0.22	21.87 ± 0.48	36.73 ± 2.91	24.39 ± 3.49	29.09 ± 0.43	25 ± 5.11	26.92 ± 0.726	25.64 ± 0.53	27.5 ± 1.3	25.64 ± 1.02
HR	1.22 ± 0.056	1.32 ± 0.003	1.28 ± 0.007	1.58 ± 0.073	1.32 ± 0.061	1.41 ± 0.008	1.33 ± 0.09	1.37 ± 0.013	1.34 ± 0.009	1.37 ± 0.024	1.34 ± 0.018

**Table 5:** Qualitative and quantitative dimensional parameters for O1–O11 formulations.

Code	Average Weight ± SD (mg)	Diameter ± SD (mm)	Average Radius (mm)	Thickness ± SD (mm)
O1	0.2017 ± 0.0059	9.060 ± 0.003	4.530	2.809 ± 0.017
O2	0.2021 ± 0.0054	9.061 ± 0.002	4.531	2.921 ± 0.036
O3	0.1981 ± 0.0076	9.041 ± 0.012	4.521	2.457 ± 0.054
O4	0.1993 ± 0.0046	9.059 ± 0.004	4.529	2.715 ± 0.086
O5	0.1993 ± 0.0059	9.122 ± 0.004	4.561	2.565 ± 0.127
O6	0.1979 ± 0.0047	9.094 ± 0.004	4.547	2.899 ± 0.005
O7	0.2044 ± 0.0049	9.080 ± 0.006	4.540	2.790 ± 0.061
O8	0.1970 ± 0.0057	9.083 ± 0.010	4.542	2.458 ± 0.123
O9	0.1991 ± 0.0053	9.037 ± 0.017	4.519	2.360 ± 0.061
O10	0.1973 ± 0.0066	9.032 ± 0.008	4.516	2.376 ± 0.058
O11	0.1993 ± 0.0045	9.076 ± 0.012	4.538	2.374 ± 0.024

**Table 6:** The CBD ODTs answers for the dependent factors.

Evaluated Parameter	Formulation Code										
Code	O1	O2	O3	O4	O5	O6	O7	O8	O9	O10	O11
Y1	0.65	0.29	0.35	0.252	0.24	0.71	0.49	0.62	0.51	0.30	0.46
Y2	44.17 ± 8.9	67.5 ± 9.2	34.67 ± 6.77	80.33 ± 11.88	19.33 ± 2.56	28.5 ± 6.05	31.17 ± 7.15	28.67 ± 10.08	26 ± 8.45	43.13 ± 3.48	53.13 ± 4.98
Y3	21.87 ± 1.86	9.6 ± 0.86	28.95 ± 5.35	8.64 ± 1.74	137.52 ± 4.95	20.84 ± 4.28	41.07 ± 2.38	88.08 ± 5.76	29.55 ± 3.04	13.82 ± 2.26	18.21 ± 2.58
Y4	4.11 ± 1.12	3.21 ± 1.24	1.15 ± 0.08	4.49 ± 1.01	7.03 ± 1.07	46.51 ± 1.12	37.65 ± 1.22	7.17 ± 1.45	64.68 ± 1.65	67.77 ± 1.78	48.92 ± 1.21
Y5	14.74 ± 1.43	7.59 ± 2.12	2.7 ± 0.12	7.92 ± 1.56	7.41 ± 1.87	68.55 ± 1.76	61.79 ± 2.11	24.68 ± 2.34	70.62 ± 2.65	76.48 ± 2.46	55.19 ± 1.34
Y6	18.21 ± 2.43	11.35 ± 3.22	6.79 ± 2.4	12.32 ± 4.1	29.09 ± 3.3	72.81 ± 2.92	62.1 ± 3.11	51.45 ± 2.14	76.33 ± 2.45	82.6 ± 3.12	75.90 ± 2.34
Y7	27.69 ± 3.21	14.71 ± 3.23	10.82 ± 2.55	14.73 ± 2.76	37.64 ± 2.76	73.07 ± 3.12	65.79 ± 3.14	64.90 ± 2.58	79.28 ± 3.45	84.12 ± 2.67	88.35 ± 2.12
Y8	44.05 ± 2.65	22.93 ± 2.54	14.62 ± 2.65	20.53 ± 3.54	46.7 ± 2.87	75.54 ± 4.06	62.68 ± 2.45	66.74 ± 2.56	81.92 ± 2.67	87.91 ± 2.78	92.14 ± 2.56
Y9	48.46 ± 3.4	30.16 ± 3.12	21.45 ± 3.21	29.55 ± 3.3	53.05 ± 3.5	76.33 ± 3.31	63.87 ± 3.21	80.16 ± 3.12	93.91 ± 3.13	92.49 ± 3.43	99.88 ± 2.89

**Table 7:** Quality of fit—Statistical parameters.

Code	R^2^ Adj	SDY	RSD	N
Y1	0.48	0.17	0.12	11
Y2	0.72	18.83	9.97	11
Y3	0.44	0.37	0.28	11
Y4	0.74	26.66	13.57	11
Y5	0.60	30.05	19.04	11
Y6	0.84	30.03	11.94	11
Y7	0.81	30.26	10.79	11
Y8	0.79	27.98	11.09	11
Y9	0.80	28.09	10.48	11

**Table 8:** The composition of the optimal formulation—O12.

Abbreviation	Mass (mg)
CBD	10
PODTG2	155.62
EMCS	7.5
SRB	6.56
MNT	5
PLX407	12.82
BFL	2.5
Final mass	200

**Table 9:** Estimated versus obtained values.

Code	Estimated Values	Experimental Values	Residuals
Y1	0.41%	0.23%	+0.18%
Y2	35 N	36.83 ± 1.67 N	−1.83 N
Y3	37.14 s	27.03 ± 1.57 s	+10.07 s
Y4	58.58%	35.93 ± 2.1%	+22.63%
Y5	68.58%	57.36 ± 3.1%	+11.22%
Y6	77.68%	87.22 ± 2.85%	−9.54%
Y7	83.62%	93.68 ± 2.32%	−10.06%
Y8	87.3%	99.25 ± 4.15%	−11.95%
Y9	94.63%	99.3 ± 6.74%	−4.67%

## Data Availability

Not applicable.

## Supplementary Materials

The following supporting information can be downloaded at: https://www.mdpi.com/article/10.3390/pharmaceutics14071467/s1, Figure S1. The chromatograms for the CBD ODT without API (A) and blank solution (B). Figure S2. Carry-over for the 10 µg/mL concentration. Figure S3. The chromatograms for LLOQ (A) and ULOQ (B). Table S1. The composition of the ODT without API. Table S2. The extended list of coefficients of tablet dependent variables.

## Abbreviations

ODT	Orodispersible Tablet
CBD	Cannabidiol
PODTG2	Prosolv^®^ ODT G2
PETsp	Prosolv^®^ EasyTab sp
CCS	Vivasol^®^ Sodium Croscarmellose
EMCS	Emcosoy^®^ STS IP
Ph. Eur.	European Pharmacopeia
FDA	Food and Drug Administration
EMA	European Medical Agency
THC	Tetrahydrocannabinol
API	Active Pharmaceutical Ingredient
QbD	Quality by design
OFAT	One factor at a time
DoE	Design of Experiments
SRB	Sorbitol
MNT	Mannitol
PLX407	Poloxamer 407
BFL	Banana flavor
ICH	International Committee of Harmonization
UHPLC	Ultra-High-Pressure Chromatography
UV-VIS	Ultraviolet-Visible
ACN	Acetonitrile
SD	Standard Deviation
USP	United States Pharmacopeia
ANOVA	Analysis of Variance
R^2^	Coefficient of determination
R^2^ adj.	The adjusted coefficient of determination
Q^2^	Variation of the response
SDY	The standard deviation of the Y
RSD	Residual Standard Deviation
N	Number of experiments
LLOQ	The lowest limit of quantification
IQCS	Intermediate quality control standard
ULOQ	The upper limit of quantification
