# Supplementary material for: Development and Evaluation of Cannabidiol Orodispersible Tablets Using a 23-Factorial Design
Source: Pharmaceutics. 2022 Jul 14;14(7):1467. doi: 10.3390/pharmaceutics14071467 (PMC9324952; doi:10.3390/pharmaceutics14071467)
Supplement: Supplementary file 1 [file pharmaceutics-14-01467-s001.zip › pharmaceutics-1784040-supplementary.pdf]

Supplementary materials

Selectivity

To verify the selectivity of an ODT without CBD, an ODT containing all components except the API was prepared. The composition can be found in **Table S1**.

Table S1. The composition of the ODT without API.

Code	Amount (mg)
PETsp	84.22
PODTG2	84.22
CCS	3.75
EMCS	3.75
SRB	6.56
MNT	5
PLX407	10
BFL	2.5

Linearity

Linearity was verified over a calibration range of 0.5 and 10 μg/mL using five concentrations. The equation obtained was y=23559x+675.69, while the coefficient of correlation was R^2=0.9997.

Specificity

The retention time in optimal condition was 2.8 min, which can be considered fast. While using a blank sample (dissolution media) or the ODT without CBD, no interferences were observed, as can be seen in **Figure S1**.

A

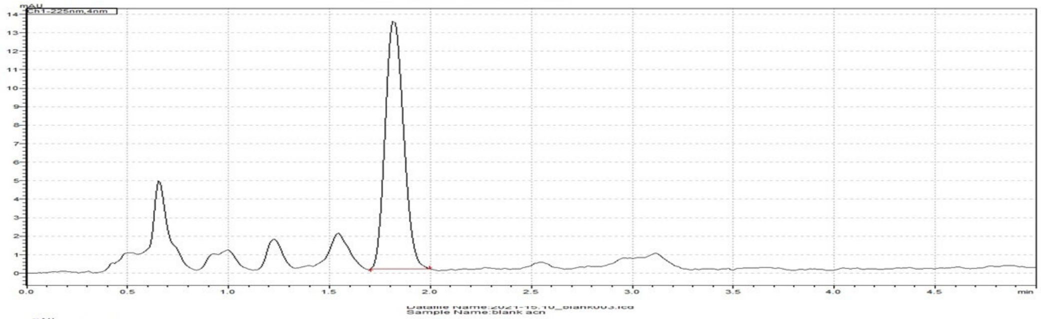

B

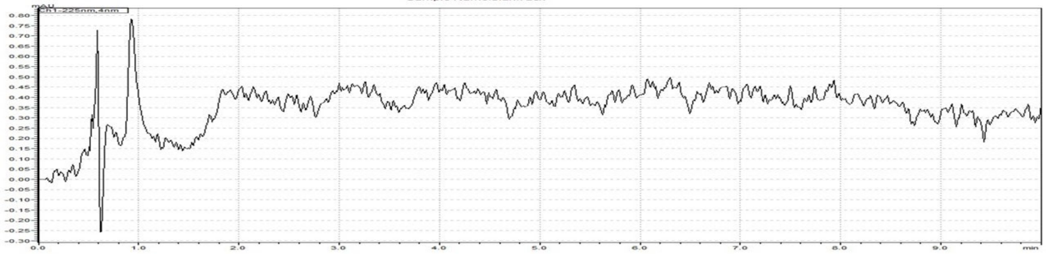

Figure S1. The chromatograms for the CBD ODT without API (A) and blank solution (B).

Carry-over

Three solutions with concentrations of 0.5 µg/ml, 2.5 µg/ml, and 10 µg/ml were injected. Before and after each solution, a blank sample consisting of dissolution media was injected. Carry-over phenomena were observed at none of the concentrations. The lack of carry-over for the 10 µg/ml concentration is presented in **Figure S2**.

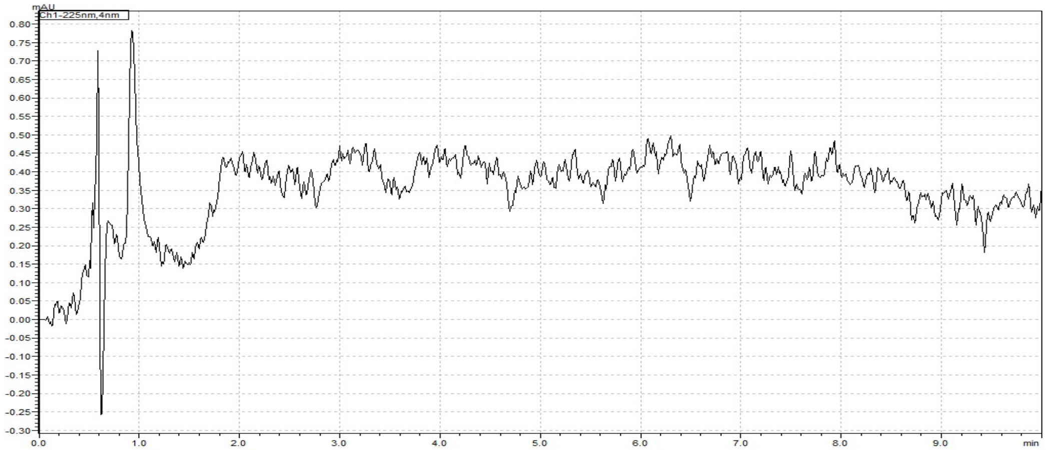

Figure S2. Carry-over for the 10 µg/ml concentration.

Accuracy and precision

To verify the precision of the method, three levels of concentrations were used to evaluate the amount of CBD released: 0.5 µg/ml, (LLOQ – lowest limit of quantification), 2.5 µg/ml (IQCS – intermediate quality control standard), and 10 µg/ml (ULOQ – the upper limit of quantification). The ULOQ was chosen taking into consideration the maximum concentration that might be obtained theoretically. The LLOQ was set at 0.5 µg/ml (5% of the ULOQ). In the pharmaceutical industry during analytical method validation, this is a value that is often used, and considering the pharmaceutical formulation that has to be developed, a high concentration of API has to be released in a short time. The coefficient of variation was between 0.56 and 0.95%, while the accuracy's coefficient of variation were in the range of 99.11-101.09%. The chromatograms for LLOQ and ULOQ are presented in **Figure S3**.

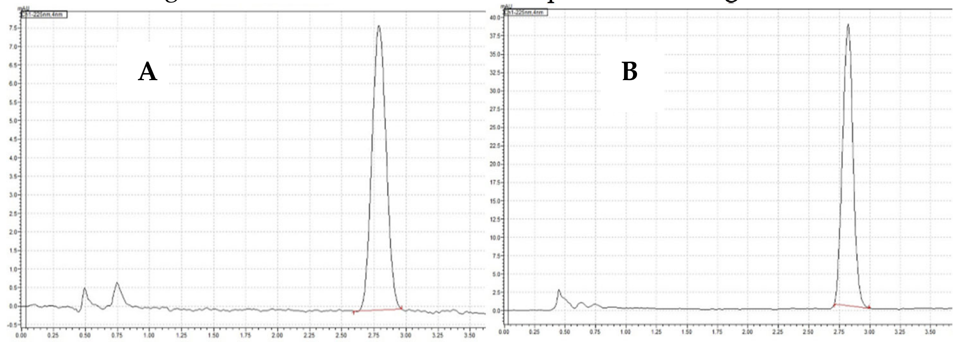

Figure S3. The chromatograms for LLOQ (A) and ULOQ (B)

Table S2. The extended list of coefficients of tablet dependent variables

Terms	Variables								
	Y1	Y2	Y3	Y4	Y5	Y6	Y7	Y8	Y9
**Intercept**	0.446	41.50	1.41	60.45	67.43	78.28	83.91	87.32	95.42
**X1 (PODTG2)**	-0.021	-7.83	0.097	-	-	-	-	-	-
**X1 (PETsp)**	0.021	7.83	-0.097	-	-	-	-	-	-
**X1 (PODTG2)* X2 (EMCS)**	-	-	-	-	-	-	-	-	-
**X1 (PODTG2)* X2 (CCS)**	-	-	-	-	-	-	-	-	-
**X1 (PETsp)* X2 (EMCS)**	-	-	-	-	-	-	-	-	-
**X1 (PODTG2)* X2 (CCS)**	-	-	-	-	-	-	-	-	-
**X2 (EMCS)**	-	-	-	-	-	-	-	-	-
**X2 (CCS)**	-	-	-	-	-	-	-	-	-
**X3**	0.025	-11.21	0.256	9.54	14.47	18.53	19.39	16.72	16.07
**X1 (PODTG2)*X3**	-0.119	7.19	-	-	-	-		-	-
**X1 (PETsp)*X3**	0.119	-7.19	-	-	-	-	-	-	-
**X2 (EMCS)*X3**	-	-	-	-	-	-	-	-	-
**X2 (CCS)*X3**	-	-	-	-	-	-	-	-	-
**X3*X3**	-	-	-	-37.23	-34.4	-36.11	-36.19	-34.48	-36.03
